# Establishment of real-time quantitative reverse transcription polymerase chain reaction assay for transcriptional analysis of duck enteritis virus UL55 gene

**DOI:** 10.1186/1743-422X-8-266

**Published:** 2011-06-01

**Authors:** Ying Wu, Anchun Cheng, Mingshu Wang, Shunchuan Zhang, Dekang Zhu, Renyong Jia, Qihui Luo, Zhengli Chen, Xiaoyue Chen

**Affiliations:** 1 Institute of Preventive Veterinary Medicine, Sichuan Agricultural University, Wenjiang, Chengdu city, Sichuan, 611130, China; 2 Avian Disease Research Center, College of Veterinary Medicine of Sichuan Agricultural University, 46 Xinkang Road, Ya'an, Sichuan 625014, China; 3 Key Laboratory of Animal Disease and Human Health of Sichuan Province, Sichuan Agricultural University, Wenjiang, Chengdu city, Sichuan, 611130, China

## Abstract

**Background:**

Real-time quantitative reverse transcription polymerase chain reaction assay (qRT-PCR) has become the benchmark for detection and quantification of target gene expression level and been utilized increasingly in detection of viral load and therapy monitoring. The dynamic transcription variation of duck enteritis virus UL55 gene during the life cycle of duck enteritis virus in infected cells has not been reported yet.

**Results:**

The newly identified duck enteritis virus UL55 gene was amplified and cloned into pMD18-T vector after digestion to generate a recombinant plasmid pMD18-T/UL55 for the establishment of qRT-PCR as standard DNA. The results of agarose gel electrophoresis and melting curve analysis demonstrated the primers we designed for qRT-PCR were specific and available. We used β-actin as a reference gene for normalization and established two standard curves based on pMD18-T/UL55 and pMD18-T/β-actin successfully. Based on that, the transcriptional analysis of DEV UL55 gene was performed, and the result suggested the expression of UL55 mRNA was at a low level from 0 to 8 h post-infection(p.i.), then accumulated quickly since 12 h p.i. and peaked at 36 h p.i., it can be detected till 60 h p.i.. Nucleic acid inhibition test was carried out for analyzing a temporal regulation condition of DEV UL55 gene, result revealed that it was sensitive to ganciclovir. Synthesis procedures of DEV UL55 gene can be inhibited by ganciclovir.

**Conclusions:**

The method we established in this paper can provide quantitative values reflecting the amounts of measured mRNA in samples. It's available for detection and quantification, also can be used in DEV diagnosis. The DEV UL55 gene was produced most abundantly during the late phase of replication in DEV-infected cells and the transcription of it depended on the synthesized DNA. DEV UL55 gene is a γ2 gene which occurs last and have a strict requirement for viral DNA synthesis.

## Background

Duck viral enteritis(DVE) is one of the most widespread and destructive diseases of waterfowls in the family Anseriformes(ducks, geese, and swans)[1]. It is an acute, contagious and lethal disease which causes substantial mortalities in both farmed and wild waterfowl. Commercial waterfowl industry had been suffering considerable economic losses since it was discovered in Netherland[2]. Duck enteritis virus(DEV), alternatively known as duck plague virus(DPV), is the causative agent of DVE and has been clustered to the family Herpesviridae determinately[3]. This vital virus tends to establish a long period of asymptomatic carrier state in waterfowl which could hardly be noticed, causes high mortality, decreased egg production of them inevitably[4]. Moreover, waterfowl recovered from this disease often turned into carriers of DEV and often shed virus. Once the latent DEV is activated, waterfowl involved in have to suffer a catastrophe.

In recent years, due to the advent of molecular biology and advancements in research related to DEV, mankind has been able to understand and control of it to a certain degree. However, the monitoring and controling stratage of regular methods always be frustrated because the hidden virus is only detectable during the intermittent shedding period of it[5]. Thus, a method which can reflect and monitor the virus proliferation dynamics, host-virus interactions, tropism, and active/latent infection will be popular.

Availability of genome sequences now provides unique opportunity for unraveling the complex molecular mechanisms of DEV infection. While identification of a genome sequence offers an insight into what its genes can do, the identification of its expression profile provides vital information on what it is doing at any given moment[6]. Gene expression levels change over time, as proteins interfere with gene transcription. Proteins and DNA interact in a complex feedback system of gene expression control, in which some proteins foster gene expression as transcription factors, while others reduce transcription activity as inhibitors. Furthermore, protein-protein interactions can increase or reduce the influence of certain proteins on transcription. These networks of gene expression control form the basis of essential cellular processes such as the cell cycle, development, and disease progression[7]. Measurement of coding and non-coding RNA in specific gene transcription processes enables discovery of new regulatory pathways[8], the injury mechanism[9], the molecular and mechanistic details of these complex events at the level of individual cell[10], validation of drug targets, and diagnosis of disease[11,12].

Quantitative measurements of expressed mRNA(Messenger RNA, the product of gene transcription) is the main way to investigate the expression of a particular gene[13]. To date, various methods that have been used to quantify mRNA of genes, including in situ hybridization techniques[14], RNase protection assays[15], Northern blotting and reverse transcription(RT)-PCR[16]. Each of them can be used alone or common used to detect specific mRNA and precisely determine transcription levels. However, quantification of target gene transcript levels by real time quantitative reverse transcriptase polymerase chain reaction(qRT-PCR) has proven to be a much poweful method due to its potential for high-throughput, together with regular introduction of enhanced or novel chemistries, more reliable instrumentation and improved protocols[17].

As we all know, virus starts its replication following infection of a cell. Genes involve in any link of it play a pivotal role in the life cycle of virus. It has been reported that the Herpes simplex virus 1 and 2(HSV-1, HSV-2) UL55 gene participates a series of biological process about viral assembly, maturation, egress, and release[18-20]. Animal herpes viruses all share some common properties. It has been speculated that the product of DEV UL55 gene plays an accessory role in the very process we have mentioned above[21]. In recent years, a method based on PCR with an automatic confirmation phase has been developed. This method, which is known as the fluorescent quantitative real-time PCR(FQ-PCR), has been used widely to quantify the number of genomic copies of DEV[22,23]. However, analysis of DEV UL55 gene expression, which promises to provide insight into the complex biological processes and therapeutic treatment haven't been reported yet.

In this paper, we constructed a recombinant plasmid pMD18-T/UL55 as standard DNA to establish a qRT-PCR for transcriptional analysis and classification of DEV UL55 gene. β-actin has been used as endogenously expressed reference genes for internal controlling[24]. Besides, we designed an inhibitive experiment to comfirm our inference by the addition of ganciclovir(virus DNA synthesis inhibitor).

## Materials and methods

### Virus and cells

DEV CHv strain(a high-virulence field strain of DEV) was obtained from Key Laboratory of Animal Diseases and Human Health of Sichuan Province. Duck embryo fibroblast(DEF) monolayer was incubated at 37°C with 5% CO_2 _in Minimal Essential Medium(MEM) supplemented with 10% fetal bovine serum(FBS), 100 U/ml penicillin, and 100 μg/ml streptomycin. For virus infection, MEM medium supplemented with 2-3% FBS was used[25]. The experimental research on animals in our text have following the national satandard <Laboratory Animal--Requirements of Environment and Housing Facilities>(GB 14925--2001) and the care of laboratory animal and the animal experimental operation have conforming <Sichuan Agricultural University Administration Rule of Laboratory Animal>.

### PCR primers design

Primers used for amplification in this paper were designed using Oligo 6.0 software and their sequences were listed in Table 1. P1 and P2 were designed for conventional PCR according to the newly identified DEV UL55 gene[GenBank: EU071034][26] and were expected to generate products of approximately 799 bp. Enzyme sites(*BamH I *and *Xho I*) were added to the primer sequences for cloning before synthesis by TaKaRa(Dalian, China). The P3, P4 primers based on the nucleotide sequence of the DEV UL55 gene were expected to produce a 124 bp fragment by real time PCR(RT-PCR). Duck house keeping gene β-actin was used as reference gene for quantification, the amplification of it was performed by RT-PCR using primers P5, P6 and supposed to generate a 178 bp fragment. The primers involved in RT-PCR were synthesized by Invitrogen(Shanghai, China) and purified by corresponding HPLC system.

**Table 1:** Sequences of primers

Name	Type	Sequences (5' to 3')	Length(nt)	Amplicon (bp)
P1	Forward	GGATCCATGGCCGACGCGAAGGCGGT	26	799
P2	Reverse	CTCGAGGCTTGGGTCTTTACTTTTTGCGCGGAAC	34	
P3	Forward	TATTCTTCTGCGGGCTCA	18	124
P4	Reverse	CATAGACGATGCTCC	15	
P5	Forward	CCGGGCATCGCTGACA	16	178
P6	Reverse	GGATTCATCATACTCCTGCTTGCT	24	

The underlined sequences of P1 and P2 are *BamH I *and *Xho I *restriction sites, respectively.

### Preparation of standard plasmid DNA templates

DNA extraction from DEV infected DEF cells was performed by using TIAN-amp Genomic DNA extracting kit(Tiangen Corporation, Beijing, China) according to the manufacture's instructions. The amplification of DEV UL55 gene was performed by conventional PCR using extracted DEV DNA as a template and the purification of it was carried out by the Gel Extraction kit according to the manufacturer's instructions. The purified product was digested with *BamH I *and *Xho I*, then ligated into the same digested pMD18-T vector(Takara, Japan) at 16°C overnight. Ligation mixtures were transformed into competent Escherichia coli DH5α cells by heat shock, following by the incubation at 37°C for 16 h in Luria-Bertani broth plates which containing 100 mg/ml ampicillin. The positive clones were designated as recombinant plasmid pMD18-T/UL55. After confirmed by PCR, restriction enzyme digestion(*BamH I *and *Xho I*) and sequencing(Dalian TAKARA Biotechnology Co.), the correct recombinant plasmids pMD18-T/UL55 were extracted as standard plasmid for qRT-PCR using the plasmid extraction kit(Tiangen Corp, Beijing, China). Smartspec3000 spectrophotometer(Bio-Rad Corp, Hercules, CA) was used to determine the concentration of pMD18-T/UL55 by measuring the absorbance at 260 nm, and the purity of it was confirmed using the 260/280 nm ratio. The recombinant plsmid pMD18-T/β-actin preserved in our laboratory was constructed in the same way and used as standard plasmid DNA templates for endogenous controling.

### Detecting the specificity of primers

Conventional PCR were carried out to detect the specificity of designed primers(P3, P4 and P5, P6) by using DEV DNA as templates. The total volume of PCR protocol was 25 μl: 12.5 μl PCR mix, 1.0 μl of each primer(20 pM each), 1.3 μl DNA template, sterile water was added into the mixture to 25 μl. Reactions were performed at 95°C for 5 min, followed by 30 cycles of 94°C for 50 s, 55.5°C for 45 s and 72°C for 1 min, at last extended at 72°C for 10 min. The amplicons were verified by 1% agarose gel electrophoresis and analyzed using gel imaging system(Bio-Rad, USA).

### Optimization of RT-PCR[17]

A few key components of real-time PCR should be optimized to achieve ideal results. Considering SYBR Green I stain method supply premix RT-PCR master mix, we just need to optimize the final concentration of primers and the annealing temperature of them. The volume of primers(concentration 20 pM) ranging from 0.5 to 1 μl and different annealing temperatures ranging from 53.4 to 63.4°C were optimized in separate PCR and readjusted to increase the efficiency of amplification. The RT-PCR was performed in an iCycler IQ Multicolor Real-Time PCR Detection System(Bio-Rad, USA) with a recommended 20 μl protocol according to the manufacturer's protocol: SYBR Green I Mix 9 μl, each of the primer 20 pM, 1 μl standard template, and finally autoclaved double-filtered nanopure water was added to get the final volume of 20 μl. Replace water as template for negative controlling(NTC). Each run consisted of initial denaturation at 95°C for 1 min following by 40 consecutive cycles of denaturation at 94°C for 30 s and different annealing temperatures for 30 s. Each reaction of DEV UL55 and β-actin were optimized in triplicate based on their primers.

### Establishment of standard curves

The determined recombinant plasmid pMD18-T/UL55 and PMD18-T/β-actin were serially diluted 10-fold in TE buffer, pH 8.0, from 10^-1 ^to 10^-7^. Each dilution of them was tested in triplicate and used as amplification templatea to construct standard curves by plotting the plasmid copy number logarithm against the Ct values under the optimum conditions. The Primers(P3, P4 and P5, P6) were used for the amplification of UL55 and β-actin by RT-PCR, respectively. Meanwhile, melting curve analysis were performed to verify the specificity of primers. The Bio-Rad iCycler IQ detection software created the standard curves and calculated the correlation coefficient of them. Positive control(standard plasmid without dilution) and negative control(NTC) were done the parallel experiment for quality controlling.

### RNA extraction and cDNA synthesis

Preparation of DEFs were carried out as we described above. To detect the transcription phase of DEV UL55 gene in infected cells in vitro, total RNA were isolated from mock-infected or DEV-infected cells at different times(0.5, 1.0, 1.5, 2.0, 3.0, 4.0, 6.0, 8.0, 12, 24, 36, 48 and 60 h p.i.) by using the Total RNA Isolation System(Takara) according to the manufacturer's instruction, and the integrity of the extracted RNA were analyzed by 1.0% agarose gel electrophoresis. The extracted cell volume equivalent amount of total RNA(10 μl) was digested with RNase-free DNase I(Takara) at 30°C for 30 min to eliminate the contamination of chromosomal DNA. After that, the DNase I was inactivated at 66°C for 15 min. After checking the purity of them by A260/A280 ratio(greater than 1.8), 100 ng RNA of each time was used as template for reverse transcription. According to the manufacturer's instruction, the purified RNA was immediately inversed transcribed to cDNA at 37°C for 1 h by Quant Reverse Transcriptase(Takara) and stored at -70°C.

### Quantitative detection of samples

The prepared cDNA of different time post infection as we mentioned above were used as templates to perform real time PCR for quantification. Using primers(P3, P4 and P5, P6) in corresponding reaction to amplify different cDNA samples as the establishment of standard curves. RT-PCR was performed in a volume of 20 μl as we optimized previously. Meanwhile, negative controls without templates(NTC) were performed in parallel. All reactions were performed in triplicate and in at least three independent reactions, and the average relative content of DEV UL55 gene transcripts was calculated using the 2-ΔΔCt method by iCycler IQ Multicolor Real-Time PCR Detection System(Bio-Rad, USA) procedure[25].

### Nucleic acid inhibition test

Nucleic acid inhibition tests were performed by adding 300 μg/ml ganciclovir(virus DNA synthesis inhibitor) into DEV infected DEFs. At the same time, infected DEFs without the addition of ganciclovir was prepared as a comparison. RNA extraction and cDNA synthesis were carried out as we described above, but the time of isolation depends on the transcription level of DEV UL55 gene we have figured out in previous experiment. Then, P3, P4 and P5, P6 were used for conventional PCR for the amplification of UL55 and β-actin gene, respectively. Each of them was performed in triplicate and in at least three independent reactions as intraassay and interassay repeatability experiments. The production of PCR were analyzed by 1.0% agarose gel electrophoresis under ultraviolet rays.

## Result

### Cloning of DEV UL55 Gene

**Figure 1:**
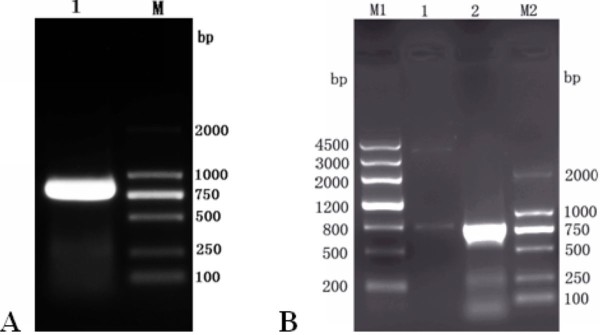
**Cloning of DEV UL55 gene and identification of recombinant plasmid pMD18-T/UL55 by restriction enzyme digestion**. **A**, The amplification of DEV UL55 gene by conventional PCR using primers P1, P2. Lane 1, the amplified product of DEV UL55 gene; M, DNA marker(DL2000). **B**, Identification of recombinant plasmid pMD18-T/UL55 by restriction enzyme digestion. Lane 1, the recombinant plasmid pMD18-T/UL55 were digested with *BamH I *and *Xho I*; Lane 2, the PCR product of the recombinant plasmid pMD18-T/UL55; M1, DNA marker III; M2, DNA marker(DL2000).

DEV UL55 gene was amplified from its genomic DNA using a couple of primers(P1, P2). Electrophoretic analysis showed a fragment of the expected size of approximate 799 bp by PCR(Figure 1A, Lane 1). After digested with *BamH I *and *Xho I *restriction enzymes, a purified fragment represented DEV UL55 gene was directionally inserted into the same digested pMD18-T vector to yeild the pMD18-T/UL55 plasmid. The obtained recombinant pMD18-T/UL55 was verified by particular restriction enzyme digestion analysis(Figure 1B, Lane 1), PCR(Figure 1B, Lane 2) and Sequencing(data not shown).

### Specificity of designed primers

**Figure 2:**
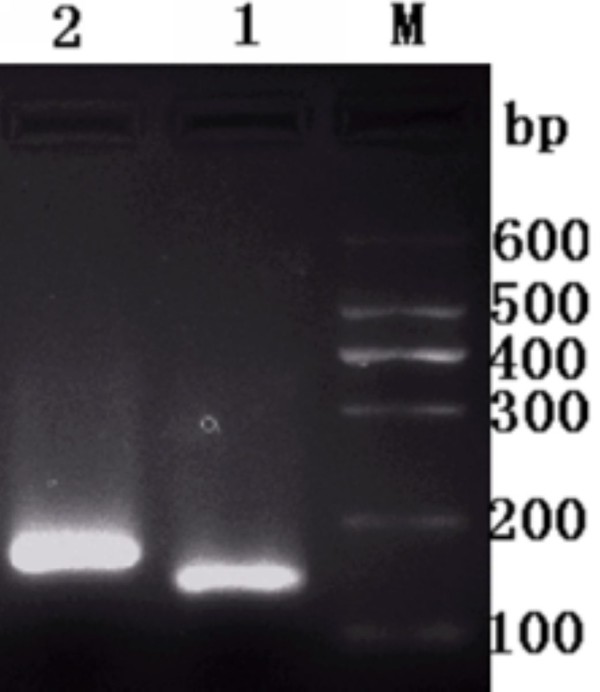
**Detecting the specificity of designed primers of DEV UL55 gene and β-actin**. M, DNA Maker I; Lane 1, the amplified product of DEV UL55 gene by conventional PCR using quantitative primers P3, P4; Lane 2, the amplified product of β-actin by conventional PCR using quantitative primers P5, P6.

The primers(P3, P4) of RT-PCR were designed for quantitative analysis according to the sequence of DEV UL55 gene. We carried out a conventional PCR based on DEV DNA to test and verify the specificity of them. Gel electrophoresis showed a band of approximate 124 bp as we expected(Figure 2, Lane 1). Meanwhile, detecting primers P5, P6 of β-actin by PCR using DEV DNA as template as well. As a result, a perspective band about 178 bp was obtained without any primer dimer and nonspecific amplification(Figure 2, Lane 2).

### Development and optimization of qRT-PCR

Primer pairs and reaction conditions were optimized using the standard pMD18-T/UL55 plasmid and pMD18-T/β-actin as templates pool prior to amplification. The optimal protocol of qRT-PCR was determined by melting curve and amplification efficiency of it. Evaluating alternatives according to the Ct value and flsorescence intensity. The minimum Ct value and upmost flsorescence value were supposed to occurrence simultaneously in an optimal protocol. Therefore, the optimized 20 μl real-time PCR reaction system could be summarized as follows: 0.5 μl(20 pM) each primers, 1 μl DNA template, 9 μl SYBR Green I Mix and 9 μl autoclaved double-filtered nanopure water. And, the optimized annealing temperature for pMD18-T/UL55 and β-actin was 55.5°C and 60.0°C, respectively. The optimized protocol was used for subsequent experiments.

### Establishment of qRT-PCR standard curves

**Figure 3:**
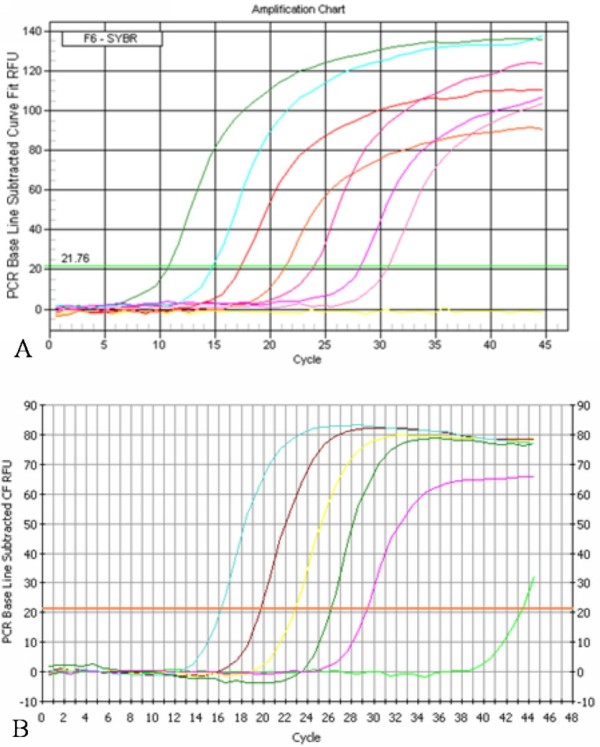
**The amplication curves of DEV UL55 gene and β-actin by qRT-PCR**. **A**, Ten-fold dilutions of standard pMD18-T/UL55 ranging from 1.0 × 10^-7 ^to 1.0 × 10^-1 ^copies/reaction were used for amplification, as indicated in the x-axis, whereas the corresponding Ct values are presented on the y-axis. **B**, Ten-fold dilutions of standard pMD18-T/β-actin ranging from 1.0 × 10^-8 ^to 1.0 × 10^-4 ^copies/reaction were used for amplification, as indicated in the x-axis, whereas the corresponding Ct values are presented on the y-axis.

**Figure 4:**
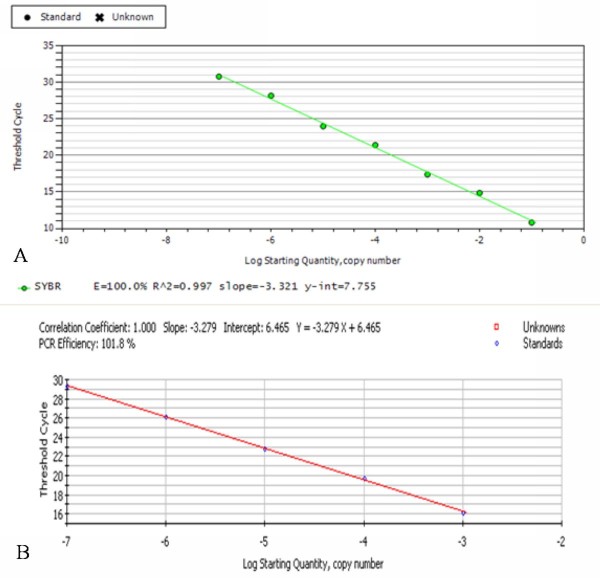
**The standard curves of DEV UL55 gene and β-actin by qRT-PCR**. **A**, The standard curve of DEV UL55 gene was calculated by iCycler IQ 5 software. Each dot represents the result of triplicate amplification of each dilution. The standard curve equation is Y = -3.321X + 7.755, the correlation coefficient and the slope value of the regression curve were calculated and indicated. **B**, The standard curve of reference gene β-actin was calculated by iCycler IQ 5 software. Each dot represents the result of triplicate amplification of each dilution. The standard curve equation is Y = -3.279X+6.465, the correlation coefficient and the slope value of the regression curve were calculated and indicated.

The amplification curves of pMD18-T/UL55 and PMD18-T/β-actin were shown in Figure 3(A, B), and the corresponding fluorescent quantitative real-time PCR standard curves of them were shown in Figure 4(A, B), These curves were generated by employing a serially dilutions of standard plasmids pMD18-T/UL55 and PMD18-T/β-actin whose copy number has been learned for qRT-PCR reaction under optimal conditions with the iCycler IQ Detection System.

The standard curve of pMD18-T/UL55(Figure 4A) displayed a clear linear relationship with a correlation coefficient[27] of 0.997 and high amplification efficiency (100%). Parameters involved were calculated automatically by the iCycler IQ software. The quantitative relation of them can be described by an equation which was capable of quantifying the amount of unknown samples: Y = -3.321X+7.755(Y = threshold cycle, X = log starting quantity).

Figure 4B ploted the standard curve of PMD18-T/β-actin through the same pathway. On the basis of the results of correlation coefficient(1.000) and PCR efficiency(101.8%), we were able to obtain the equation:Y = -3.279X+6.465(Y = threshold cycle, X = log starting quantity).

**Figure 5:**
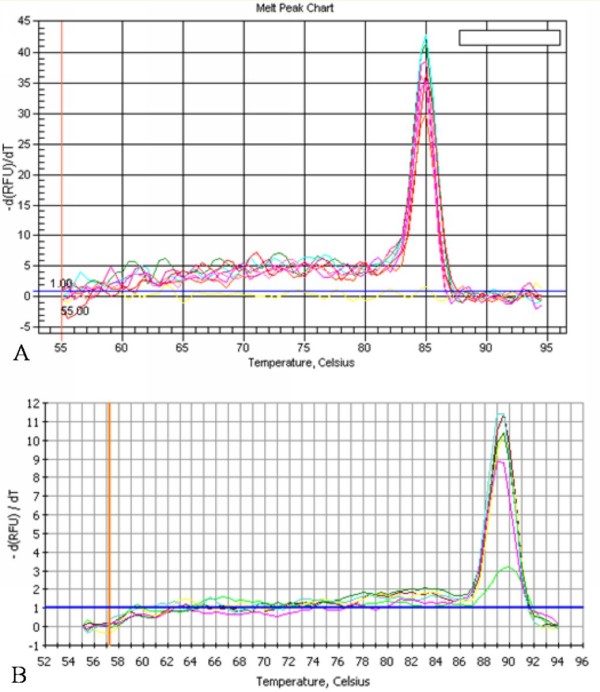
**The melt curve of DEV UL55 gene and β-actin by qRT-PCR**. **A**, The melt curve of DEV UL55 gene displayed a single peak at 85.0°C. **B**, The melt curve of β-actin displayed a single peak at 89.5°C.

Melt curves of them were shown in Figure 5A and Figure 5B, respectively. As a result, both of them presented a single peak. The consequences of melting curve were consistent with a single reation product for each sample, that supported the highly specificity of primers in another aspect.

### Integrity and purity analysis of extracted RNA

**Figure 6:**
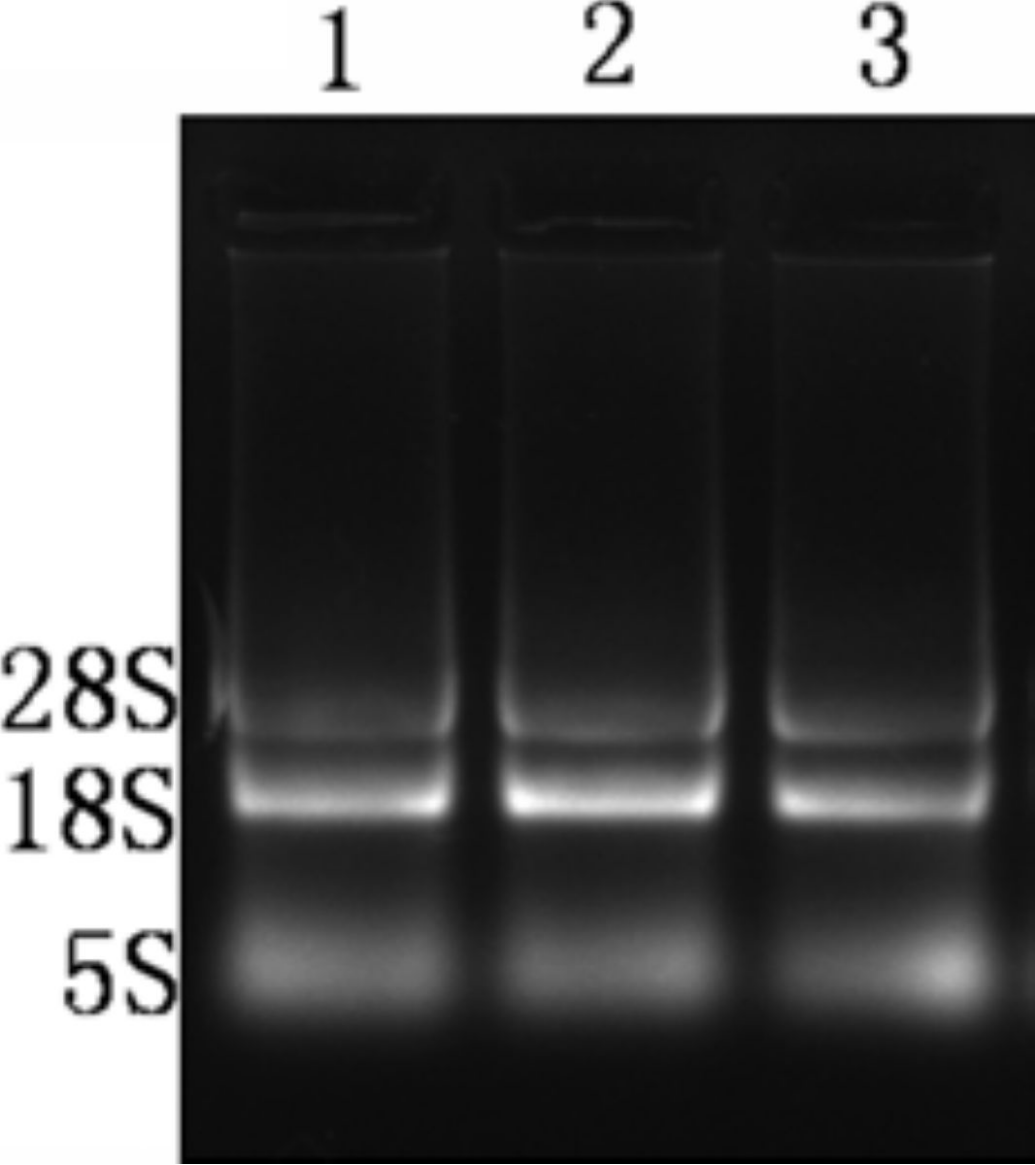
**Verification of extracted RNA by 1%agarose gel electrophoresis**. Total RNA isolated from mock-infected and DEV-in-fected cells at various times post-infection were analysed using 1.0% agarose gel electrophoresis.

The total RNA isolated from mock-infected and DEV infected cells were verified by 1.0% agarose gel electrophoresis. As a result, Figure 6 displayed three major bands: 28S rRNA band(located at approximately 5 Kb), 18S rRNA band(located at approximately 2.0Kb) and 5S rRNA band. The 18S band was approximately twice the intensity of the 5S band. The purity of extracted RNA were determined by OD260/OD280, results suggested we have extracted RNA of high-quality since they were basically ranged from 1.8 to 2.0(data not shown).

### Transcriptional analysis of DEV UL55 gene in infected cells in vitro by established qRT-PCR

**Figure 7:**
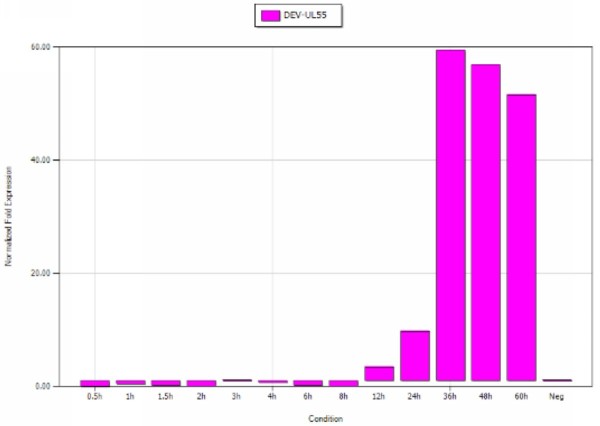
**Transcriptional analysis of DEV UL55 gene in infected cells in vitro**. The transcriptional analysis of DEV UL55 in infected DEFs were performed by qRT-PCR. The transcriptional expression of DEV UL55 gene was normalized to a reference gene(β-actin) and relative to NTC control. The average relative content of the DEV UL55 gene transcripts were calculated at 0.5, 1.0, 1.5, 2.0, 3.0, 4.0, 6.0, 8.0, 12, 16, 24, 36, 48, and 60 h post-infection using iCycler IQ 5.

The dynamic expression of DEV UL55 gene were analyzed on total RNA by qRT-PCR with SYBR Green I and reverse transcription PCR. The average relative content of DEV UL55 gene transcripts were calculated using the 2-ΔΔCt method by a procedure of iCycler IQ 5 software. As shown in Figure 7, the relative expression level of UL55 gene was at a low level in the first 8 h post-infection(p.i.). After that period, the transcripts of UL55 gene became obviously detectable at 12 h p.i. and then accumulated markedly until reached a peak at 36 h p.i., then declining slowly thereafter but maintaining a high level of transcription when we detected at 60 h p.i. However, the transcripts of DEV UL55 gene were not detected in mock-infected DEFs. Each of the sample was detected in triplicate. The samples were amplified by the primers of the reference gene β-actin for internal control.

### Nucleic acid inhibition test

**Figure 8:**
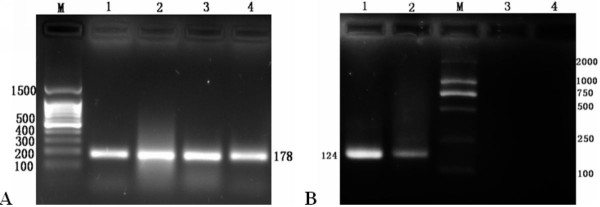
**Nucleic acid inhibition test**. **A**, M, 100 bp DNA ladder; Lane 1, Lane 2, transcriptional analysis of β-actin in infected DEFs at 24 h and 36 h p.i., respectively. Lane 3, Lane 4, transcriptional analysis of β-actin in infected DEFs by the addition of nucleic acid inhibitor ganciclovir at 24 h and 36 h p.i., respectively. **B**, M, DNA maker(DL2000); Lane 1, Lane 2, transcriptional analysis of DEV UL55 gene in infected DEFs at 36 h and 24 h p.i., respectively. Lane 3, Lane 4, transcriptional analysis of DEV UL55 gene in infected DEFs by the addition of nucleic acid inhibitor ganciclovir at 24 h and 36 h p.i., respectively.

Based on previously transcription analysis of DEV UL55, we extracted total RNA at 24 h and 36 h p.i. and reversely transcribed them to cDNA. They were amplified by conventional PCR using primers of β-actin(P5, P6) and DEV UL55(P3, P4), respectively. The cataphoresis results were shown in Figure 8, from the electrophoretograms we got prospective bands both at the 24 h p.i. and 36 h p.i. of β-actin(Figure 8A, Lane 1 and Lane 2) and DEV UL55(Figure 8B, Lane 2 and Lane 1). Besides, we also found visible bands of β-actin at 24 h p.i. and 36 h p.i. with nucleic acid inhibitors(Figure 8A, Lane 3 and Lane 4). However, either of the inhibitor additive representive lane in Figure 8B displayed a visible band which probably means no product have been amplified(Figure 8B, Lane 3 and Lane 4). In other words, the DNA synthesis inhibitor ganciclovir restrained the virus replication cycle. By the way, the results of intraassay and interassay repeatability experiments indicated a high degree of coherence which make the result more reliable.

## Discussion

In this study, we have eastablished a real-time quantitative reverse transcription polymerase chain reaction assay(qRT-PCR) based on DEV UL55 gene successfully. It is a powerful tool for quantitative analysis of target RNA due to its high-throughput, together with excellent sensitivity and specificity, low contamination risk, ease of performance and speed, more reliable instrumentation and improved protocols. The application of it in quantitative analysis of DEV UL55 gene samples has demonstrated the transcription of UL55 gene occures in the last stage of viral replication cycle.

Reverse transcription combined with real time PCR has been created as qRT-PCR, which has become the standard for the detection and quantification of RNA targets and firmly established as a mainstream research technology due to the outstanding advantages[11]. To date, the technology has been used vastly in fields of pathogeny detection(virus, bacterium, fungus, etc), gene expression analysis(cell factor, growth factor, transcription factor, etc) and so on[28,29]. To our knowledge, viruses complete their composition in host cell through the expression and replication of viral genome. Gene expression mechanisms of them in a cell act as both an "on/off switch" to direct which genes are expressed, and as a "volume control" in regulating gene expression. The monitoring of gene expression will be a useful maker of disease progression and as a component of studies into the efficacy of antiviral compounds[30]. Real time quantitative PCR became an attractive method to study gene expression because of its low inter-assay and intra-assay variablilty and its equivalent or greater analytical sensitivity in comparison with traditional method. The quantification of templates by qRT-PCR can be performed in two ways: as absolute quantification and as relative quantification. The former should be performed in situations where it is necessary to determine the exact transcript copy number of targets present in the sample. Nevertheless, the latter describes the change in expression of the target gene relative to some reference group such as an untreated control or a sample at time zero in a time-course study[31]. Thus, we employed relative quantification as an ideal method to study dynamic transcription profiling of DEV UL55 gene in this paper.

In theory, qRT-PCR differs from PCR mainly by the addition of a preliminary step, the initial conversion of RNA into a DNA template by an RNA-dependent DNA polymerase(reverse transcriptase). In fact, this additional procedure results in a much more fragile and variable assay[10,32]. Expression levels of target genes measured by real-time RT-PCR should be normalized by comparison with transcript abundances of reference genes to.avoid serious distortion of experimental results[33]. β-actin occurs in all duck nucleated cell types would be universally valid due to a constant expression level regardless of the expremental conditions[34,35]. Results demonstrated the introduction of it has corrected the sample-to-sample variation of UL55 which might occur at a number of stages throughout the experimental protocol of RNA quantification experiments[32] and affect efficiencies of the reverse transcription(RT) and polymerase chain reaction(PCR) reactions[36]. Besides, mock infected DEFs were introduced as homologous controls for alternative normalization. They were amplified with UL55 gene using the same PCR primers and have eliminated the introduced contamination during our experiment as we expected.

The principle of qRT-PCR assays is straightforward: following the RT of RNA into cDNA, it requires a suitable detection chemistry to report the presence of PCR products, an instrument to monitor the amplification in real time and appropriate software for quantitative analysis[11]. Detection chemistries can be either probe- or non-probe based. Although the specificity of non-probe based chemistries depends on the specificity of the primers, an important advantage of coverting the optimized conventional RT-PCR assays immediately into real-time assays has made it to be an ideal method for quantification. The most widely used non-probe-based chemistry detects the binding of SYBR Green I to ds(double-stranded) DNA[37]. SYBR Green binds to the minor groove of dsDNA, causing fluorescence signals to increase synchronously during PCR as DNA products increase exponentially[38]. Using SYBR Green I for quantification of DEV UL55 gene has eliminated the need for complicated probe design. However, optimization of PCR conditions was supposed to be carried out prior to the use of SYBR Green I to reduce the effect of the nonspecific binding to PCR artifacts such as primer dimers that may contribute to the fluorescence signal. The single band generated by coventional PCR as we expected has demonstrated the the primers we designed for UL55 gene and β-atin were specific and availabe for amplification. Also, melting curve analysis of UL55 gene and β-atin comfirmed the conclusion of primers as the single peak of each curve presented in our research. Meanwhile, the optimized protocol was determined by the way. All these ensured the protocol has a low potential to form secondary structures, including self and crosshybridization with other oligonucleotides in the PCR.

Quantification can be relative to an external standard curve based on the use of a dilution series of an external standard, which can be used to generate a standard curve of Ct(threshold cycle) against initial target copy number[30]. In our reaserch, the plasmids pMD18-T/UL55 and pMD18-T/β-actin were particularly constructed to generate standard curves. The copy numbers of unknown samples can be calculated from the linear regression of that standard curve, with the log(nanograms) input amount of RNA in each well as the x values and Ct cycle as the y values[39]. The most important parameters Ct was defined as the cycle when sample fluorescence exceeds a chosen threshold above calculated background fluorescence. The determination of it depends upon the sensitivity and ability of the instrument to discriminate specific fluorescence from background noise, the concentration and nature of the fluorescence-generating component and the amount of template initially present[30]. The more templates present at the beginning of the reaction, the fewer cycles it takes to reach a point in which the fluorescent signal is first recorded as statistically significant above background[40]. Since background fluorescence is not a constant or absolute value but is influenced by changing reaction conditions, the value of a Ct recorded for a particular sample might vary with the variation of background fluorescence varies. In order to avoid false positive results, we runned samples in triplicate with NTC(no templates control) as negative control which is too low to cross the default threshold level. If the corrections results of a negative control becoming positive, it makes the result unbelievable. Moreover, it has been reported that the NTC recording a Ct less than 30 suggested the presence of high levels of contamination in the laboratory[10]. After caculation, the standard curves of pMD18-T/UL55 and pMD18-T/β-actin were described by the equation: Y = -3.321X + 7.755 and Y = -3.279 + 6.465, respectively. The Ct value and the correlation of established standard curves demonstrated an excellent linear relationship of them, and the method we established can be used for the detection of UL55 gene in an extensive boundary. In addition, the amplification efficiencies of pMD18-T/UL55(100%) and reference pMD18-T/β-actin(101.8%) were quite close to each other. It was crucial because it affects the accuracy of any calculated expression result directly[11,41].

It has been reported that the transcription of herpesvirus genes were processed by stages: the expression of immediate-early(IE) genes occured first, following by the expression of early(E) genes and late(L) genes sequentially[42]. In order to figure out the type of DEV UL55 gene and the transcription profiling of it, as well as how it changes over time, we collected triplicate samples of cells at different time. The dynamic proliferation of UL55 gene calculated by iCycler IQ 5 suggested the transcription of DEV UL55 gene became detectable at 8 h p.i. compare to negative control, peaked at 36 h, then decreased but kept a high level until 60 h p.i. etc. According to the gene expression time series terminology, DEV UL55 gene could be clustered to late genes, which was consistent with the report of HSV-1, HSV-2[18] UL55 gene. That probably means the product of DEV UL55 gene may execute the similary function of late genes in DEV life cycle such as HSV UL55 gene did[43]. It has been reported that late genes predominantly encode proteins to form the virion particle[44]. Packaging of the viral particles including viral assembly, maturation, egress, and release which mostly occurred at the later stages of DEV life. DEV UL55 gene started transcription late in host cells and kept a comparatively high leve during our detection can be the resonable interpretation of UL55 gene production participating in the above procedures.

Late genes are subdivided into two categories as leaky-late(γ1) or strictlate(γ2). The γ1 genes can be suboptimally expressed in the absence of viral DNA synthesis, whereas the γ2, have a strict requirement for viral DNA synthesis[42]. Thus, we dealt the infected DEF cells with some canonical medicine to analyze a temporal regulation condition for UL55 gene. Studies of vulnerable patient populations of HSV have indicated that daily use of antivirals such as acyclovir and valacyclovir can reduce reactivation rates due to the interference with viral replication. Evaluation of effection of antivirus drug used for DEV in vitro proved acyclovir had a good inhibitory activity against DEV. Based on the unavailable phosphonoacetate and acyclovir in reality, we replaced it with ganciclovir as an alternative which has the same inhibition for DNA synthesis. Results in our study indicated DEV UL55 gene was sensitive to ganciclovir since the synthesis procedures of DNA have been inhibited by corresponding inhibitor. In other words, that implied DEV UL55 gene actually is a γ2 gene whose transcription was mainly depends on the DNA which has been synthesized previously.

## Conclusions

The established qRT-PCR in our research possesses a lot of advantages such as highly amplification efficiency, extensive linear range of Ct value, short detection period and high throughput(96 samples can be detected at one time) etc. The dynamic variation rule of DEV UL55 gene based on this quantification method provides an accurate and sensitive index for DEV life period judgement which was significant for DEV diagnosis and treatment. Besides, the determination of regimentation sort of DEV UL55 is helpful for functional research of DEV UL55 gene which are quite insufficiency and contributes to the pathopoiesia mechanism of DEV. Furthermore, the sensitive speciality of UL55 gene to antivirus drug and the established qRT-PCR can be supposed to be a candidate for evaluation of drug effection for DEV treatment.

## Competing interests

The authors declare that they have no competing interests.

## Authors' contributions

YW carried out most of the experiments and drafted the manuscript. ACC, MSW, SCZ, DKZ, RYJ, QHL, ZLC and XYC helped in experiments and drafted the manuscript. All authors read and approved the final manuscript.
